# Comparative Analysis of Plasmids in the Genus *Listeria*

**DOI:** 10.1371/journal.pone.0012511

**Published:** 2010-09-02

**Authors:** Carsten Kuenne, Sonja Voget, Jordan Pischimarov, Sebastian Oehm, Alexander Goesmann, Rolf Daniel, Torsten Hain, Trinad Chakraborty

**Affiliations:** 1 Institute of Medical Microbiology, Justus-Liebig University, Giessen, Germany; 2 Goettingen Genomics Laboratory, Institute for Microbiology and Genetics, Georg-August University Goettingen, Goettingen, Germany; 3 Bioinformatics Resource Facility, Center for Biotechnology, Bielefeld University, Bielefeld, Germany; University of Hyderabad, India

## Abstract

**Background:**

We sequenced four plasmids of the genus *Listeria*, including two novel plasmids from *L. monocytogenes* serotype 1/2c and 7 strains as well as one from the species *L. grayi*. A comparative analysis in conjunction with 10 published *Listeria* plasmids revealed a common evolutionary background.

**Principal Findings:**

All analysed plasmids share a common replicon-type related to theta-replicating plasmid pAMbeta1. Nonetheless plasmids could be broadly divided into two distinct groups based on replicon diversity and the genetic content of the respective plasmid groups. *Listeria* plasmids are characterized by the presence of a large number of diverse mobile genetic elements and a commonly occurring translesion DNA polymerase both of which have probably contributed to the evolution of these plasmids. We detected small non-coding RNAs on some plasmids that were homologous to those present on the chromosome of *L. monocytogenes* EGD-e. Multiple genes involved in heavy metal resistance (cadmium, copper, arsenite) as well as multidrug efflux (MDR, SMR, MATE) were detected on all listerial plasmids. These factors promote bacterial growth and survival in the environment and may have been acquired as a result of selective pressure due to the use of disinfectants in food processing environments. MDR efflux pumps have also recently been shown to promote transport of cyclic diadenosine monophosphate (c-di-AMP) as a secreted molecule able to trigger a cytosolic host immune response following infection.

**Conclusions:**

The comparative analysis of 14 plasmids of genus *Listeria* implied the existence of a common ancestor. Ubiquitously-occurring MDR genes on plasmids and their role in listerial infection now deserve further attention.

## Introduction

The genus *Listeria* comprises six non-pathogenic species *L. marthii, L. innocua*, *L. welshimeri*, *L. seeligeri*, *L. grayi,* and *L. rocourtiae*, and two species with pathogenic potential viz. *L. monocytogenes* and *L. ivanovii*, which can cause both human and animal infections [1]–[7]. Since *L. monocytogenes* exhibits resistance towards heat and cold stress it can proliferate in food processing environments [8] and thus colonize dairy and meat products which have caused several outbreaks as well as sporadic cases of listeriosis [9]. Three serotypes of the species *L. monocytogenes* viz. 1/2a, 1/2b and 4b are responsible for 95% of all human clinical infections [10].

Extrachromosomal DNA was previously detected in many *L. monocytogenes* wildtype strains with rates of isolation ranging from 0–79% with an overall average of 30% [11]–[15]. Two studies which examined 173 [14] and 322 [16] isolates of *L. monocytogenes* respectively found an overrepresentation of plasmids in strains from food and the environment in comparison to those obtained from clinical cases. It was shown that plasmids were found more frequently (75%) in recurrent *L. monocytogenes* strains sampled from food/processing environments than in those from sporadic strains (35%) [17]. Plasmids were also more frequently associated with serogroup 1 strains compared to those from serogroup 4. It was determined that 95% of the *L. monocytogenes* plasmid-positive strains were resistant towards cadmium versus only 13% of the plasmid-negative strains [14] and that the *cadAC* genes were similar to those previously detected in *Staphylococcus aureus* [18]. Only in two cases antibiotic resistance of *L. monocytogenes* could be traced to a plasmid [19], [20]. Plasmids were also previously described for *L. innocua* [2] and *L. grayi* [12]. Furthermore plasmids pAMbeta1 and pIP501 of *Streptococcus* could be transferred to *L. monocytogenes* where they stably replicated underlining the broad-host range of these replicons and their potential for horizontal transfer between strains of these genera [12], [21]. The contribution of plasmids to the infectious process has not been examined and their evolutionary history is not yet well understood apart from homologies to other gram-positive plasmids such as with plasmid pXO2 from *Bacillus anthracis* which is required for the pathogenic properties of this species [22]–[24].

## Results and Discussion

### *Listeria* plasmids overview

We determined the entire sequences of plasmids from *L. monocytogenes* 7 UG1 SLCC2482, *L. monocytogenes* 1/2c UG1 SLCC2372, *L. monocytogenes* 1/2b UG1 SLCC2755 and *L. grayi* subspecies *grayi* UG1 DSM20601. For comparative analysis we included sequences of the plasmids pLM33 of *L. monocytogenes* Lm1, pCT100 of *L. monocytogenes* DRDC8, pLM80 of *L. monocytogenes* H7858, pLM5578 of *L. monocytogenes* 08-5578 and pLI100 of *L. innocua* Clip11262 which were downloaded from the NCBI website as well as further five gapped *L. monocytogenes* plasmid sequences from strains FSL J1.194, FSL R2-503, FSL N1-017, FSL F2-515 and J0161 which were retrieved from the Broad Institute (http://www.broad.mit.edu) database. All plasmid contigs were remapped and reannotated.

It should be noted that plasmids sequenced by the Broad Institute were found to contain a large number of SNPs leading to truncated genes. A recent study assumed that higher selective pressure was responsible for this phenomenon [23], but other studies with this data have also indicated truncations in many essential housekeeping genes on the chromosomes of these strains [25] indicating an alternative explanation i.e. sequencing errors. Indeed the average sizes of coding sequences from *L. monocytogenes* plasmids sequenced in this study vary between 260 and 264 while those obtained from the Broad study range from 131 to 245 amino acids, respectively (Table 1).

**Table 1:** General features of 14 plasmids of genus *Listeria*.

Host	Plasmid	Isolation	Status	Length [bp]	ORFs^a^	MGEs^b^	Mean Number of Amino Acids per CDS	Source/Accession
*L. monocytogenes* 1/2b Lm1	pLM33	cheese	closed	32307	36	9	258	GU244485
*L. monocytogenes* 1/2a FSL F2-515	pF2-515	meat	contigs (11)	37163	61	12	131	Broad Institute^c^
*L. monocytogenes* 7 UG1 SLCC2482	pLM7UG1	human	closed	50100	55	13	260	FR667690
*L. monocytogenes* 1/2c UG1 SLCC2372	pLM1-2cUG1	human	closed	50100	54	13	264	FR667691
*L. monocytogenes* 1/2b FSL J1.194	pJ1-194	human	contigs (1)	57536	69	16	223	Broad Institute^c^
*L. monocytogenes* 1/2b UG1 SLCC2755	pLM1-2bUG1	human	closed	57780	63	16	261	FR667692
*L. monocytogenes* 1/2b FSL R2-503	pR2-503	human	contigs (3)	56540	86	20	159	Broad Institute^c^
*L. monocytogenes* 4b FSL N1-017	pN1-017	trout	contigs (3)	56037	62	13	245	Broad Institute^c^
*L. monocytogenes* 1/2a 08-5578	pLM5578	human	closed	77054	76	11	291	CP001603
*L. monocytogenes* 1/2a J0161	pLMJ0161	human	contigs (2)	82700	90	10	266	Broad Institute^c^
*L. monocytogenes* 4b H7858	pLM80	meat	contigs (2)	81588	88	11	264	AADR01000010, AADR01000058
*L. grayi* subspecies *grayi* UG1 DSM20601	pLGUG1	chinchilla	closed	79249	99	8	224	FR667693
*L. innocua* 6a Clip11262	pLI100	cheese	closed	81905	84	24	273	AL592102
*L. monocytogenes* 4 DRDC8	pCT100	milk	closed	37279	34	6	292	U15554

a Open Reading Frames.

b Mobile Genetic Elements.

c http://www.broad.mit.edu/annotation/genome/listeria_group.

Plasmid length does not include spacers which were inserted between contigs. All genes automatically predicted by GenDB [58] to encode a recombinase, transposase, integrase, invertase or resolvase are denoted as mobile genetic element (MGE).

Since it is not feasible to include locus tags for up to 14 homologs of a gene we decided to only include a gene name or annotation in the text which can be used in conjunction with a homology matrix (Table S1) to identify the respective loci. Furthermore a public Geco server [26] including all plasmids of this study as well as their reference annotations was set up (http://bioinfo.mikrobio.med.uni-giessen.de/geco2plasmids/).

### Phylogenetic clustering based on replication protein

All plasmids contained a similar minimal replicon consisting of three genes necessary for replication (*repA*) and partitioning (*repB, repC*) as well as the origin of replication [27]–[32] and a gene encoding a DNA polymerase IV. The replicon is a member of the pAMbeta1 family of theta-replicating plasmids and its proteins are most closely related to plasmids from the genera *Bacillus* (pXO2, pAW63, pBT9727), *Streptococcus* (pSM19035) and *Enterococcus* (pRE25, pVEF1, pVEF2) with protein identities ranging from between 36–56%. An exception to this homology was found to be RepC which shows no sequence similarity but a similar location, size and orientation as its putative functional homologs in the plasmids of the aforementioned genera. The genes encoding *repB/*C are overlapping indicating an operon. Interestingly, the translesion DNA polymerase has previously been suggested to stimulate spontaneous deletions during DNA repair [33], [34] and could thus contribute to variation and adaptation of both plasmid and host genes when present.

**Figure 1:**
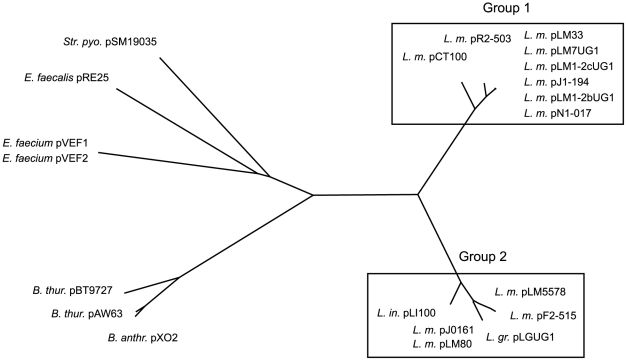
Phylogenetic tree of the replication initiation protein. Phylogenetic tree based on the replication initiation protein RepA of plasmids of genus *Listeria* and related genera. In the case of pF2-515 and pN1-017 multiple proteins had to be merged due to premature stopcodons. Clustalw [35] was used to create the multiple sequence alignment which was visualized using Dendroscope [60]. The clustering of the replication initiation proteins shows a clear separation into two phylogenetic groups.

To examine the relationship of the plasmid backbones we joined all fragments of the RepA proteins and used CLUSTALW [35] to create a phylogenetic tree (Figure 1). This methodology clearly confirms the relatedness of these plasmids to those present in other gram-positive strains and separated the plasmids of genus *Listeria* into two distinct phylogenetic groups consisting of *L. monocytogenes* serotypes 7, 1/2c, 1/2b, 4b FSL N1-017 and serogroup 4 DRDC8 in one cluster and *L. monocytogenes* serotype 1/2a, 4b H7858, *L. innocua* and *L. grayi* in the other. The plasmid of *L. monocytogenes* 1/2b strain F2-515 was an exception to this since it clustered with group 2 confirming observations from a previous study [23]. Plasmid sizes generally correlate with the clustering based on the replication initiation protein ranging from 32–57 kb in group 1 and 77–83 kb in group 2 (Table 1), again with the exception of F2-515 which belongs to group 2 but has a size similar to plasmids of group 1.

### Comparative genetic analysis

**Figure 2:**
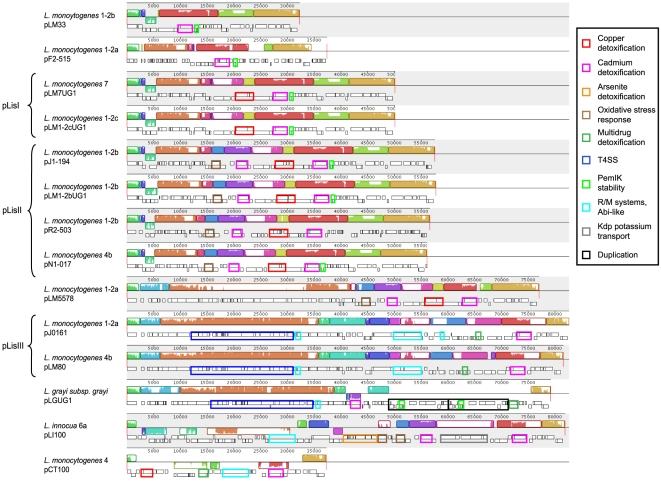
Complete alignment of plasmid sequences. Whole-sequence alignment of 14 plasmids of genus *Listeria* as computed by Mauve [59]. Relevant loci are marked specifically. These include genes involved in heavy metal detoxification (copper, cadmium, arsenite), multidrug resistance (MDR: SMR, MATE), phage defense (R/M systems, Abi), oxidative stress response, an incomplete type IV secretion system (T4SS), a PemIK stable inheritance module, a Kdp-type potassium transport system and a sequence duplication. It should be noted that Mauve was not able to identify all homologies due to algorithmic limitations.

The replicon-based distinction is mirrored by the gene content to some extent, which indicates a highly similar set of genes for most plasmids of group 1, with a more heterogenous distribution for group 2 (Figure 2). Genes were considered homologs if BlastP found a sequence identity of at least 30% covering more than 80% of both proteins (Table S2). Apart from the replicon no other feature is conserved overall, but all plasmids contain a cadmium resistance operon (*cadA/C*) [18] with the exception of pLGUG1 which lacks *cadC* and harbors a transposase at that relative position.

Apart from pCT100, plasmids of group 1 are closely related to each other and mainly differ by two putative indel events. Most of them can be grouped into two types which will be referred to as pLisI (pLM7UG1, pLM1-2cUG1) and pLisII (pJ1-194, pR2-503, pLM1-2bUG1, pN1-017). The smallest plasmid pLM33 was previously described to contain multiple transposases, remnants of *Listeria* phage A006, CRISPR associated protein Cas5 implied in phage defense and a Clp protease related to *Lactobacillus* which is involved in environmental stress response [23]. Despite clustering with group 2 according to its rep-protein, the sequence of plasmid pF2-515 shows a much higher homology to plasmids of group 1 which is not reflected by its gene-content due to a large number of false stop codons leading to a computed average protein length of only 131 amino acids. The indel between pLM33 and pLisI is 18 kb in size and contains multiple transposases, a copper-transporting P-type ATPase and a multicopper oxidase (MCO) implied in copper detoxification [36]. Either one or both copper resistance genes show putatively premature stopcodons in pLM7UG1, pJ1-194, pR2-503 and pN1-017. All plasmids excepting the smallest plasmids, pLM33, pF2-515 and pCT100 contained a protein with a FIC domain (filamentation induced by cyclic adenosine monophosphate) which is implicated in the disruption of cellular functions following transfer to the host cell cytoplasm during infection [37]. The plasmids of type pLisI and pLisII differ by 6 kb. This region encodes a transposase, a cadmium-transporting ATPase as well as an NADH peroxidase and a periplasmic component of an ABC-type glycine/betaine transport system closely related to *Aerococcus viridans* ATCC 11563 with an identity of 97% and 55% respectively. NADH peroxidases are described as being necessary for decomposition of hydrogen peroxide accumulated during aerobic growth [38]. This could play a role in intracellular survival against hydrogen peroxide stress [39] as well as in defense against disinfectants which are also known to induce general and oxidative stress responses [40]. All plasmids of group 1 apart from pCT100, and pLGUG1 of group 2, carry the sequence of the PemIK toxin/antitoxin stable maintenance system described for *Lactobacillus salivarius* UCC118 plasmid pSF118–20 [41]. In most cases one of the genes (pLM7UG1, pLM1-2cUG1, pJ1-194, pLM1-2bUG1, pR2-503) could not be identified by the gene prediction, which implies a decay of this functionality in those strains.

Only half of the sequence of pCT100 is shared with any other plasmid of the genus *Listeria*, including the replicon, a cadmium resistance system, a copper transporter and an insertion of 6 kb shared with pLI100 related to 12 kb plasmid pEW104 of *Lactococcus lactis subsp. cremoris* W10. Sequences present in this 6 kb fragment include two genes probably involved in replication and a single-gene type I restriction and modification (R/M) system called *Lla*GI which was shown to confer decreased bacteriophage sensitivity to its host [42]. The other half of pCT100 consists of multiple genes indicated in copper detoxification and a Na^+^-driven multidrug efflux pump which belongs to the MATE family (multidrug and toxic compound extrusion) [43].

All plasmids of group 2 excepting pLGUG1 showed extensive homology to plasmids of group 1 further indicating a common ancestor. Plasmids pLM5578 and pLI100 share 76% and 45% nucleotide identity with pLisII respectively, with only 18% identity for the plasmids pJ0161 and pLM80. The latter two plasmids are closely related to each other and will be referred to as type pLisIII. Both contain a 40 kb region that is similar to plasmid pXO2 from *B. anthracis* which can also be found in pLGUG1 and to some extent in pLM5578 which is thought to include an incomplete type IV secretion system [22]. This system was shown to be insufficient for conjugation in pXO2 [31]. They also share a locus of 20 kb containing 24 genes including multiple transposases and two restriction modifications systems, one of them related to type III R/M system LlaFl of *Lactococcus lactis* [44]. This locus also harbors a gene encoding a triphenylmethane reductase described for the degradation of toxic synthetic dyes in *Citrobacter* [45] and a system of two genes *ebrAB*, which create a heterodimer channel involved in multidrug efflux in *Bacillus subtilis* [46]. The latter system belongs to the small multidrug resistance family (SMR) and is implied in resistance towards ethidium bromide and quarternary ammonium compounds [47] commonly found in disinfectants and could support persistence in food processing environments. Plasmid pLMJ0161 contains a specific insertion of a gene encoding an Abi-like protein implied in phage resistance [48]. The second half of pLGUG1 harbors a specific insertion spanning 24 kb which consists of a duplicated sequence comprising 17 genes most of them hypothetical. In addition this locus includes a Tn552-family transposase and a PemI/PemK post segregational killing system. Other pLGUG1 specific genes encode a MATE family multidrug efflux pump distantly related to the one found in pCT100. In pLI100 regions homologous to pLisII are interrupted by multiple pLI100-specific insertions. One of these consists of six genes related to potassium transport which span a region of 10 kb and are located on the same strand implying an operon. This Kdp-ATPase system [49] consists of a two-component signal transduction system (*kdpD/E*) and a potassium-transporting ATPase (*kdpA/B/C*). It is widely distributed among bacteria and archeae and plays a vital role in osmotic adaptation and pH regulation [50]. A homologue of this system exists in the chromosome of all fully sequenced strains of genus *Listeria* (data not shown) and contributes to growth during osmotic stress and low temperature in *L. monocytogenes* [51]. Another pLI100-specific insertion is an arsenite resistance operon related to integrative conjugative element ICESde3396 of *Streptococcus dysgalactiae subsp. equisimilis* strain NS3396 (EU142041) spanning 12 kb and consisting of seven genes which may contribute to the survival of *L. innocua* in the environment. Adjacent to this region a coenzyme A disulfide reductase gene was identified which is implied in oxidative stress response in *Borrelia burgdorferi* bb0728 [52].

In general, the plasmids of genus *Listeria* harbor a large and diverse number of mobile genetic elements. Between 6 and 24 genes per plasmid were annotated as transposase, resolvase, integrase, recombinase or invertase. This suggests that plasmids may act as an evolutionary sink for mobile genetic elements which may have shaped the diversity and evolution of plasmids in the genus *Listeria*.

### Small non-coding RNA

Recently small non-coding RNAs (sRNAs) have become a focus of research because of their roles in bacterial regulatory mechanisms [53]. Interestingly we could identify multiple putative sRNAs on listerial plasmids. One class of RNA was already described for plasmids where they are predominantly implied in replication control, segregation and conjugation [54]. To identify additional sRNAs a software called sRNAdb (J. Pischimarov, unpublished), which employs BlastN, was used to find sequence similarities previously described in *L. monocytogenes*. Using a cutoff of 60% identity and 80% coverage, four putative sRNAs [55] were identified on plasmids of genus *Listeria* (Table S2). These comprise two pairs of homologues being *rli28/rli50* and *rli44/rli46*. Homologous sequences to *rli28/rli50* could be identified in pLM80-like, pLI100 and pCT100 while *rli44/rli46* could be found in pLM33, pF2-515, pLisII, pLisIII and pLGUG1.

### Conclusion

Here we report on the completion of four new plasmid sequences, including two novel plasmids from *L. monocytogenes* serotype 1/2c and 7 strains as well as one from the species *L. grayi*. In the comparative analysis presented here we compared sequences of 14 plasmids from three species using additional sequences either previously published or deposited in databases. We found that all plasmids share a common replicon-type related to theta-replicating plasmid pAMbeta1 [56] implying a common ancestor. Nonetheless a phylogenetic division must have occurred when considering the replication initiation protein. This division was mostly mirrored by the genetic content which showed clear distinctions between those groups apart from two atypical plasmids (pF2-515, pCT100). Based on regions of synteny, we are able to trace diversification and evolution driven by indels that account for the range of plasmid sizes detected. The presence of a commonly occurring translesion repair DNA polymerase on all plasmids suggests a mechanism by which genetic deletions are generated. Since plasmids of genus *Listeria* are related to *Bacillus*, *Enterococcus* and *Streptococcus* and were described to be transferable between some of these genera [12], [21], it is likely that exchange amongst these bacteria takes place in many different environmental niches e.g. gut and soil. Also, the unexpected detection of a large number of mobile genetics elements present on these plasmids imply that these could be involved in increasing genetic diversity or even altering gene expression both at chromosomal and episomal sites. Furthermore we found multiple independent systems involved in defense against phages (type I and III restriction systems, Abi-like) in group 2 implying a role for plasmids in the dissemination of these genes to ward off bacteriophage infection. The detection of small non-coding RNAs on a number of plasmids that were homologous to those present on the chromosome of *L. monocytogenes* EGD-e suggests that sRNAs might be transfered via plasmid conjugation.

The overrepresentation of plasmids in studies examining strains from food and the environment [14], [16] and in recurrent *L. monocytogenes* strains sampled from food/processing facilities [17] is an intriguing observation. However, the presence of multiple genes involved in heavy metal resistance (cadmium, copper, arsenite) as well as multidrug efflux (MDR, SMR, MATE) and oxidative stress response (peroxidase, reductase) on listerial plasmids could assist survival and their presence may have resulted from selective pressure due to the use of disinfectants in food processing environments. Finally, we note that MDR efflux pumps have recently been shown to promote cyclic diadenosine monophosphate (c-di-AMP) as a secreted molecule able to trigger the cytosolic host response following infection [57]. The implication for the presence of MDR genes on plasmids and their role in listerial infection now deserves further scrutiny.

### Availability

Four plasmid sequences from this article have been deposited in the EMBL/GenBank database under accession numbers FR667690 (pLM7UG1), FR667691 (pLM1-2cUG1), FR667692 (pLM1-2bUG1) and FR667693 (pLGUG1). An EMBL-formatted version of all plasmids can be downloaded (http://bioinfo.mikrobio.med.uni-giessen.de/publications/listeria_plasmids/listeria_plasmids_embl.tar.gz). The data can also be compared and retrieved using Geco (http://bioinfo.mikrobio.med.uni-giessen.de/geco2plasmids/).

## Materials and Methods

### Public data sources

Contigs of five gapped *L. monocytogenes* plasmids were downloaded from the homepage of the Broad Institute (http://www.broad.mit.edu/annotation/genome/listeria_group) originating from strains FSL J1.194 (2.44), FSL R2-503 (2.52, 2.53, 2.54), FSL N1-017 (2.75, 2.76, 2.77), FSL F2-515 (2.1405, 2.1406, 2.1407, 2.1408, 2.1409, 2.1410, 2.1411, 2.1412, 2.1413, 2.1414, 2.1415) and J0161 (1.50, 1.51). The plasmids pLM33 of *L. monocytogenes* Lm1 (GU244485), pCT100 of *L. monocytogenes* DRDC8 (U15554), pLM80 of *L. monocytogenes* H7858 (AADR01000010, AADR01000058), pLM5578 of *L. monocytogenes* 08-5578 (CP001603) and pLI100 of *L. innocua* Clip11262 (AL592102) were downloaded from the GenBank database (http://www.ncbi.nlm.nih.gov/genbank/index.html).

### Isolation and sequencing

The remaining strains from *L. monocytogenes* 7 UG1 SLCC2482, *L. monocytogenes* 1/2c UG1 SLCC2372, *L. monocytogenes* 1/2b UG1 SLCC2755 and *L. grayi subspecies grayi* UG1 DSM20601 were isolated using Epicentre's MasterPure gram-positive DNA purification kit as recommended by the manufacturer. The DNA was sequenced on a 454 GS-FLX System to coverages between 16–57x. The resulting reads were assembled *de novo* with the 454 Newbler assembler and mapped vs. published plasmids to identify homologous contigs. PCR-based techniques were used to close the remaining gaps which were sequenced with Sanger ABI Big Dye technology. The sequencing was performed by Roche (Germany), Goettingen Genomics Laboratory (Goettingen, Germany) and Agowa (Berlin, Germany).

### Bioinformatics

All contigs of gapped plasmids were scaffolded according to finished plasmids and joined to a consecutive sequence using the spacer “nnnnnttaattaattaannnnn” to prevent the gene prediction from crossing contig borders. All sequences were then reordered to a putative origin adjacent to the replication initiation gene (*repA*) as described for the homolog replicon of *B. anthr*acis plasmid pXO2 [29] and automatically annotated using the GenDB system [58]. The annotation was corrected based on a comparative syntheny analysis as offered by Geco [26]. In order to compute a phylogenetic tree Clustalw [35] was applied on replication initiation proteins using standard parameters. A multiple sequence alignment of the complete plasmid sequences was created with the Mauve software [59] using a progressive alignment including seed families to increase sensitivity. Mauve was not able to identify all homologies correctly with any combination of parameters. The chosen alignment is the optimal result considering false positives/negatives (data not shown).

## Supporting Information

Table S1 This matrix shows a single-linkage clustering of all proteins of 14 plasmids of the genus *Listeria* using a minimum of 30% amino acid identity and 80% coverage. Annotation was included from the first protein of each cluster starting from the left. Clusters were sorted according to their size to ensure that mutually conserved proteins can be found at the top of the list while specific ones are moved to the bottom.
(0.07 MB XLS)

Table S2 Using BlastN with a cutoff of 60% identity and 80% coverage four sRNAs of Listeria monocytogenes EGD-e [55] could be identified on various plasmids.
(0.02 MB XLS)
